# *Pinus halepensis* Essential Oil Ameliorates Aβ1-42-Induced Brain Injury by Diminishing Anxiety, Oxidative Stress, and Neuroinflammation in Rats

**DOI:** 10.3390/biomedicines10092300

**Published:** 2022-09-16

**Authors:** Paula Alexandra Postu, Marius Mihasan, Dragos Lucian Gorgan, Fatima Zahra Sadiki, Mostafa El Idrissi, Lucian Hritcu

**Affiliations:** 1 Center for Fundamental Research and Experimental Development in Translation Medicine–TRANSCEND, Regional Institute of Oncology, 7000483 Iasi, Romania; 2 Department of Biology, Faculty of Biology, Alexandru Ioan Cuza University of Iasi, 700506 Iasi, Romania; 3 Laboratory of Molecular Chemistry and Natural Substances, Department of Chemistry, Faculty of Sciences of Meknes, Moulay Ismail University, Meknès 11201, Morocco

**Keywords:** amyloid beta peptide, *Pinus halepensis*, essential oil, anxiety, depression, Alzheimer’s disease

## Abstract

The *Pinus* L. genus comprises around 250 species, being popular worldwide for their medicinal and aromatic properties. The present study aimed to evaluate the *P. halepensis* Mill. essential oil (PNO) in an Alzheimer’s disease (AD) environment as an anxiolytic and antidepressant agent. The AD-like symptoms were induced in Wistar male rats by intracerebroventricular administration of amyloid beta1-42 (Aβ1-42), and PNO (1% and 3%) was delivered to Aβ1-42 pre-treated rats via inhalation route for 21 consecutive days, 30 min before behavioral assessments. The obtained results indicate PNO’s potential to relieve anxious–depressive features and to restore redox imbalance in the rats exhibiting AD-like neuropsychiatric impairments. Moreover, PNO presented beneficial effects against neuroinflammation and neuroapoptosis in the Aβ1-42 rat AD model.

## 1. Introduction

Ranked as the fifth-leading cause of death worldwide [1], Alzheimer’s disease (AD) currently affects approximately 50 million individuals. It is predicted that the number of AD subjects will triple by 2050 [2,3]. Although the social and psychological burden remains difficult to quantify [4], the financial burden associated with AD is staggering, reaching $818 billion in 2015 [5] and being expected to increase to more than $2 trillion by 2030 [6]. This global health crisis is deepened by the lack of medication that can delay or prevent cognitive decline [7] and the lack of a single diagnostic test able to accurately detect the disease in an early phase, clinical diagnosis of AD remains a costly and laborious process [8]. Although it relies on a complex battery of neuropsychological and neuroimaging tests [9], clinical AD diagnosis still has a sensitivity of only 81% and a specificity of 70% [10,11] as compared to the gold standard, namely pathology at autopsy [12,13].

AD is a pathologically heterogeneous and biologically multilayered disease characterized by gradual memory loss and cognitive and behavioral dysfunctions [14], currently being re-conceptualized as a biological and clinical continuum [15,16,17] that extends from a long asymptomatic phase with evidence of AD pathology but normal cognitive function, to minor cognitive changes and, ultimately, reaching to a clinically symptomatic AD phase [18]. Although mainly considered a cognitive disorder, AD is often associated with neuropsychiatric disabilities, which manifest throughout different phases of the disease continuum [19]. AD patients may exhibit a plethora of neuropsychiatric impairments, greatly fluctuating in severity and frequency, including apathy, depression, anxiety, irritability, sleep disturbances, eating abnormalities, agitation, elation, hallucinations, delusions, motor disturbances, and disinhibition [20,21,22,23]. After apathy, depression and anxiety are the most common non-cognitive impairments in AD patients [24] with prevalence rates up to approximately 40% [25], and both appearing as risk factors [26,27], early signals, or resultant symptoms of the disease [28,29,30]. Often, depressed AD patients experience more prominent difficulties with concentration and indecisiveness and higher rates of psychomotor agitation and fatigue [31], suggesting that depressive behaviors in AD patients rely on neuroanatomical substrates, such as global cerebral atrophy in gray matter volume and cortical thinning in frontal, temporal, parietal, occipital, and insular lobes [32,33]. Neuroanatomical changes were also observed in AD patients with concomitant anxiety, consisting of amygdala atrophy, thinning of the entorhinal cortex, and decreased gray matter volume in the right precuneus, inferior parietal lobule, left parahippocampal gyrus, posterior cingulate gyrus, left insula, and bilateral putamen lobes [34,35], which translates into increased irritability, restlessness, and panic attacks [36]. Moreover, in recent years, different mechanistic links underlying the coexistence of AD and anxiety, or AD and depression have been uncovered. Exacerbated inflammatory background [37,38], unbalanced oxidant–antioxidant status [39], decreased neurotrophin levels [40,41,42], impairments in neurotransmitter systems [43], and elevated levels of neurofibrillary tangles (NFTs) [44,45] appear as common ground for AD and depression, while the co-occurrence of AD and anxiety is consistent with elevated Aβ build-up [46,47] and extensive NFTs burden [48].

The occurrence of depression and anxiety in AD significantly affects the patient’s quality of life and function levels [49]. Even though various scales are used to detect depression and anxiety in AD patients [50,51], they both are still underdiagnosed and, therefore, undertreated in the AD context, most likely due to the absence of consistent diagnostic criteria [50,52]. Moreover, the general lack of known symptom-specific biology for the AD neuropsychiatric impairments raises difficulties in targeting these behaviors with agents unique to AD [53]. Therefore, depression and anxiety in AD are managed with therapeutic agents conventionally used to address major depressive disorders and generalized anxiety disorders [29,54]. However, most of the pharmacological options available for the management of AD-related neuropsychiatric impairments lack strong evidence from randomized clinical trials validating their effectiveness [55,56], generating a critical need to design and/or identify novel selective and more effective therapies.

Medicinal plants represent a rich source of a valuable therapeutic biomolecule with the potential for ameliorating anxious–depressive symptoms [57], and have been proved effective through different preclinical and clinical trials [58,59]. A randomized controlled clinical trial involving 54 AD patients showed that aromatic inhalation (one hour/day) for 3 months resulted in reduced neuropsychiatric impairments, as well as decreased oxidative stress and level inflammatory markers [60]. Hence, olfactory stimulation via inhalation might be a suitable delivery method of the essential oils to AD patients due to its low invasiveness and ease of implementation.

For *Pinus halepensis*, which appears as a potent medicinal plant with antioxidant [61,62], anti-inflammatory [63], and anti-acetylcholinesterase activities [64] and hippocampal dependent-memory enhancing properties [65], the antianxiety and antidepressant activities have not yet been evaluated. Taking into account the chemical composition of *P. halepensis* essential oil (PNO) [65], this study attempts to demonstrate the anxiety–depressive symptom-relieving properties of PNO in an AD Aβ1-42-induced rat model.

## 2. Materials and Methods

### 2.1. Production and Analysis of Essential Oil

The PNO extraction procedure and the subsequent analysis of the obtained natural product have been previously described elsewhere [65].

### 2.2. Animals

For the present study, a total of 70 three-month-old Wistar albino male rats, weighing 300 ± 15 g, were used. All the experimental animals have been accommodated in 1500 U Polysulfone cages (480 × 325 × 210 mm) with free access to water and standard certified rodent food, being housed in an artificial, ventilated, and thermally controlled room (22 °C) with a 12-h light/dark cycle (starting at 8:00 h in the morning). All of the in vivo experimental protocols have been established in agreement with the European Communities Council Directive (Directive 2010/63/EU) as well as the “Principles of Laboratory Animal Care” (NIH publication No. 85-23) regarding the protection of animals used for scientific and experimental purposes with approval of the Ethics Committee on Animal Research of the Alexandru Ioan Cuza University of Iasi, Faculty of Biology (Iasi, Romania) under license no. 15309/22 July 2019.

### 2.3. Experimental Design

Three days before the surgical interventions, the rats were randomly allocated to 7 experimental groups (*n* = 10): (1) the untreated and untested group (naive); (2) the saline-treated group (sham-operated); (3) the group treated solely with Aβ1-42 (Aβ1-42); (4) the Aβ1-42 group also receiving diazepam (Diaz) (Aβ1-42 + Diaz); (5) the Aβ1-42 group also receiving imipramine (Imp) (Aβ1-42 + Imp); (6) the Aβ1-42 group also receiving 1% *P. halepensis* essential oil (Aβ1-42 + 1% PNO), and (7) the Aβ1-42 group also receiving 3% *P. halepensis* essential oil (Aβ1-42 + 3% PNO).

### 2.4. Drug Administration

All the therapeutics used within this study were administrated via three main delivery routes.

#### 2.4.1. Intracerebroventricular (i.c.v.) Route

**Figure 1:**
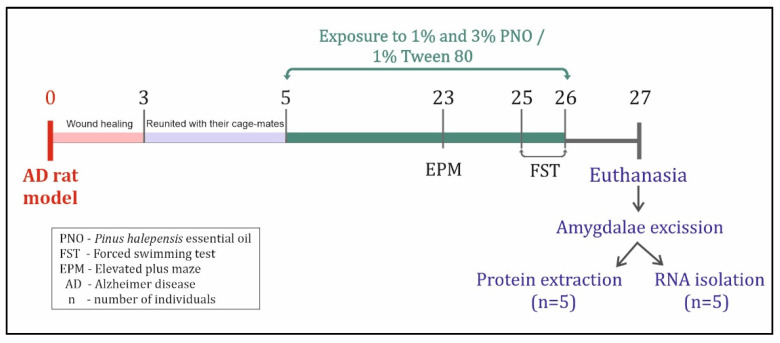
The experimental timeline. Drug administration, behavioral assessments, and tissue collection. AD, Alzheimer’s disease; EPM, elevated plus maze test; FST, forced swimming test; PNO, *Pinus halepensis* essential oil.

The i.c.v. route has been used to establish the amyloid-beta feature of AD. Through stereotaxic surgery, 50 rats received 4 μL of Aβ1-42 suspension (1 mM, Sigma-Aldrich, Darmstadt, Germany) in the third cerebral ventricle via a well-established, previously described protocol [66]. The remaining 10 rats, corresponding to the sham group, received the same volume of saline following the same protocol. Immediately after surgery, the rats were individually housed for 3 days to allow post-surgical incision healing (Figure 1), following to be regrouped with their cage-mates the next day.

#### 2.4.2. Intraperitoneal (i.p.) Route

The i.p. route has been used to deliver imipramine (Imp, 20 mg/kg) [67] and diazepam (Diaz, 3 mg/kg) [68] to the animals. Both drugs were administered to the Aβ1-42 treated rats 30 min before behavioral assessments (Figure 1) by using insulin-sized syringes.

#### 2.4.3. Inhalation Sessions

Two Plexiglass chambers (50 × 40 × 28 cm) were used for administration via inhalation by using an electronic vaporizer (Oregon Scientific WS113, Tualatin, OR, USA), placed at the bottom of the inhalation chambers. In one of the chambers, the rats corresponding to sham-operated, Aβ1-42, Aβ1-42 + Diaz, and Aβ1-42 + Imp groups were exposed to a 1% Tween 80 solution used for the dilution of the essential oils (200 μL/inhalation session). The other chamber has been used to expose the rats corresponding to Aβ1-42 + 1% PNO and Aβ1-42 + 3% PNO groups to PNO in concentrations of 1% and 3%, respectively (200 μL/inhalation session). The inhalation sessions were performed for 21 consecutive days, starting with day 5 poststereotaxic surgery and, on the test days, they were conducted 30 min before behavioral assessments (Figure 1). Each inhalation session had a duration of 15 min.

### 2.5. Behavioral Analysis

#### 2.5.1. Elevated plus Maze Test

Anxiety-like behavior has been assessed by using the elevated plus maze test (EPM) developed by Pellow et al. [69]. A black Plexiglass apparatus (Coulbourne Instruments, Allentown, PA, USA) formed of a central sheath raised 50 cm above the surface, with two opposing closed arms (49 × 10 cm) and two opposing open arms have been used for each animal testing. The rats were centrally positioned within the maze, oriented to the same open arm, and allowed to explore for 5 min. An observer recorded the following parameters: the time spent in the open and closed arms and the entries made on the open and closed arms [70]. Before testing each rat, the maze was thoroughly cleaned with cotton and 10% ethanol solution and completely dried with paper towels. All the assessments were conducted in a phonically isolated room.

#### 2.5.2. Forced Swimming Test

Depressive-like behavior has been assessed by using the forced swimming test (FST) developed by Porsolt [71], with some modifications [72]. The rats were placed in a transparent cylindrical glass tank (height = 58 cm, internal diameter = 30 cm), filled with water (23 ± 1 °C) up to 30 cm. The rats were monitored throughout two swimming sessions: a 15-min pretest session, followed by a 5-min test session. The two swimming sessions were conducted 24 h apart, and two behavior forms were monitored: immobility (the lack of motion of the whole body, with small movements sufficient to keep the animal’s head above the water) and swimming (large forepaw movements, more than necessary to keep the head above the water) [73]. The tank water was changed after each tested rat. Moreover, after each swimming session, each rat was wiped with cotton towels and kept in a warm cage until the fur of the animal was fully dry.

### 2.6. Animal Euthanasia and Tissue Collection

After behavioral assessments, all animals were euthanized by using a sodium pentobarbital overdose (150 mg/kg b.w., i.p., Sigma-Aldrich, Darmstadt, Germany), humanely decapitated, and whole brains were collected. From five randomly selected rats per group, the brain regions containing amygdalas were carefully excised on ice, washed with ice-cold PBS (Santa Cruz Biotechnology, Inc., Dallas, TX, USA) for blood removal, and stored at −20 °C until the protein extraction procedure was applied (Figure 1). From the remaining five rats per group, the isolated brain regions containing the amygdala were rapidly immersed in RNA Save solution (Biological Industries, Kibbutz Beit-Haemek, Israel) and kept at −80 °C until the ribonucleic acid (RNA) extraction procedure was applied (Figure 1).

### 2.7. Biochemical Parameters Assay

#### 2.7.1. Protein Extraction

The amygdala tissue samples were removed from −20 °C, slowly thawed on ice, weighed, and homogenized (1:10) in ice-cold 0.1 M potassium phosphate buffer (pH 7.4), containing 1.15% KCl. The amygdala homogenates were centrifuged at 960× *g* for 15 min and the supernatants containing proteins were collected. For the obtained amygdala protein extracts, the total protein content was determined via a bicinchoninic acid (BCA) protein assay kit (Sigma-Aldrich, Darmstadt, Germany) as previously described [74]. The amygdala protein extracts were further used for oxidative stress-related biochemical determinations and DNA fragmentation evaluation.

#### 2.7.2. Catalase Activity Assessment

For catalase (CAT, EC 1.11.1.6) activity determination, a reaction mixture containing 150 μL phosphate buffer (0.01 M, pH 7.4) and 100 μL amygdala protein extract was completed by the addition of 250 μL 0.16 M of a hydrogen peroxide substrate solution [75]. The reaction was allowed to develop for 1 min at 37 °C and it was stopped by the addition of 1 mL of dichromate: acetic acid reagent. The control samples were prepared in the same manner, the amygdala protein extract being substituted with the same volume of phosphate buffer (pH 7.4). Subsequently, all reaction mixtures were incubated for 15 min at 95 °C, then slowly cooled to room temperature and the green color developed during the reaction was read at 570 nm on a spectrophotometer. The activity of the enzyme has been expressed as μmol of H_2_O_2_ consumed/min/mg protein.

#### 2.7.3. Superoxide Dismutase Activity Assessment

To determine superoxide dismutase (SOD, EC 1.15.1.1) activity, each 1.375 mL reaction mixture containing 1150 μL phosphate buffer (pH 7.8), 100 μL 0.1 M disodium EDTA (pH 7.8), 50 μL 1.5 mM NBT, 25 μL 0.12 mM riboflavin, and 50 μL amygdala protein extract was incubated at room temperature, monitoring the reduction of NBT to bluish-black formazan [76]. To obtain the control samples, the same protocol was followed, but the amygdala protein extract was replaced with potassium phosphate buffer. After color development, the reaction mixtures were determined spectrophotometrically at a wavelength of 560 nm, and the enzyme activity is expressed in units/mg protein.

#### 2.7.4. Glutathione Peroxidase Activity Assessment

For the glutathione peroxidase (GPX, E.C. 1.11.1.9) activity assessment, [77] each reaction mixture consisting of 475 µL of sodium phosphate buffer 0.25 M (pH 7.4), 36 µL of EDTA 25 mM, 36 µL NaN_3_ 0.4 M, and 78 µL amygdala protein extract was incubated for 10 min at 37 °C. Each reaction mixture was completed by the addition of 50 µL of GSH 50 mM and 36 µL of H_2_O_2_ 50 mM, which was followed by a secondary incubation step at 37 °C for 10 min. Finally, to each reaction mixture, 730 µL metaphosphoric acid 7% has been added and the reaction tubes were centrifuged at 14,000 rpm for 10 min. A total of 100 µL supernatant per reaction mixture has been mixed with 1270 µL disodium phosphate solution 0.3 M and 136 µL DTNB 0.04%, and the developed yellow color was read at 412 nm on a spectrophotometer. Control samples were prepared in the same manner, except that the amygdala protein extract was replaced with an equal volume of sodium phosphate buffer 0.25 M (pH 7.4). The GPX enzyme activity has been expressed as units/mg protein, where a GPX unit was defined as the amount of enzyme necessary to oxidize 1 µmol GSH/min.

#### 2.7.5. The Total Content of Reduced Glutathione Assessment

For evaluation of reduced glutathione (GSH) content [78,79], each reaction mixture contained 1.1 mL of 0.25 M sodium phosphate buffer (pH 7.4), 130 μL DTNB 0.04%, and 200 μL amygdala protein extract. Up to 1.5 mL of the reaction mixture was completed with double distilled water and the developed yellow color was read at 412 nm by using a spectrophotometer. Control samples were prepared by using the same method, with the replacement of the amygdala protein extract with the same volume of double distilled water. The total content of reduced glutathione has been expressed as μg GSH/μg protein.

#### 2.7.6. Protein Carbonyl Level Assessment

For determination of the carbonyl protein level [80], 1 mg of total protein from the amygdala protein extract was precipitated with 450 μL 20% trichloroacetic acid and centrifuged for 5 min at 960× *g*. The obtained protein deposits were incubated with 500 μL 10 mM DNPH in 2 M hydrochloric acid for 60 min at 30 °C and stirred at 5-min intervals. Subsequently, 500 μL of 20% trichloroacetic acid was added to each protein deposit. After centrifugation at 960× *g* for 5 min, three washing steps with 1 mL ethanol:ethyl acetate (1:1) were performed. Next, the protein deposits were dried at room temperature and dissolved overnight in a 6-M guanidine hydrochloride solution in 20 mM monopotassium phosphate. The extinction of the resulting reaction mixtures was determined spectrophotometrically at 370 nm and expressed as nmol DNPH/mg protein.

#### 2.7.7. Malondialdehyde Level Assessment

For the assessment of the malondialdehyde (MDA) level [81], 650 μL of 0.37% thiobarbituric acid:6.4% perchloric acid (2:1) was added to a volume of 200 μL of amygdala protein extract. The resulting reaction mixtures were incubated for 60 min at 95 °C, cooled slowly, and centrifuged for 10 min at 960× *g*. For the control samples, the amygdala protein extracts were replaced with the same volume of phosphate buffer (pH 7.0). Finally, the reaction mixtures were spectrophotometrically determined at 532 nm, and the MDA level was expressed as nmol/mg protein.

### 2.8. DNA Fragmentation Assay

The relative quantification of histone-associated DNA fragments was performed by using a cell death detection ELISA kit version 8 (Roche Diagnostics, Mannheim, Germany) as previously described [66]. The wells were first coated with antibodies directed against DNA and histone, and then the amygdala protein extracts were added. This step is followed by horseradish peroxidase-conjugated anti-DNA antibody incubation. Ten minutes after the addition of the ABTS substrate, the peroxidase retained in the immunocomplex has been quantified by using a microplate reader at a 405-nm wavelength. The enrichment factor was represented as absorbance of the sample/absorbance of the negative control. The amygdala protein extracts of healthy, untreated, and untested male rats were considered the negative controls.

### 2.9. RNA Isolation and Amygdala Real-Time Quantitative PCR (qRT-PCR)

The amygdala tissue samples preserved in RNA Save solution were removed from −80 °C, allowed to thaw at 4 °C, weighed, and homogenized in RNA lysis buffer. Total RNA was isolated from from the obtained amygdala homogenates by using the SV Total RNA Isolation System kit (Promega, Madison, WI, USA), respecting the protocol provided by the manufacturer and methods previously described [82]. Reverse transcription and real-time PCR amplification were carried out by using a GoTaq^®^ 1-Step RT-qPCR System (Promega, Madison, WI, USA) on a Rotor-Gene 6000 thermocycler (Corbett, CA, USA), according to the manufacturer’s protocol. The absolute quantification for the transcripts of interest, ARC and IL1*β*, involved primers with the following sequences: IL-1*β* forward and reverse primer (F: 5′-AGC ACC TTC TTT TCC TTC ATC TT-3′, R: 5′-CAG ACA GCA GGC ATT TT-3′, 144 bp product size) (PrimerDesign, Chandler’s Ford, UK), and ARC forward and reverse primer (F: 5′-CCC TGC AGC CCA AGT TCA AG-3′, R: 5′-GAA GGC TCA GCT GCCT GCTC-3′, 114 bp product size) (Integrated DNA Technologies, Leuven, Belgium). Data acquisition and analysis were performed by Rotor-Gene Q-Pure Detection Software v. 2.2.3. (Qiagen, CA, USA).

### 2.10. Statistical Analysis

GraphPad Prism v9.1.0 software (La Jolla, CA, USA) has been used for statistical data analysis. One-way analysis of variance (ANOVA), followed by Tukey’s posthoc multiple comparison test was applied, and all data is expressed as the mean ± standard error of the mean (S.E.M.). The statistical significance was set at *p* < 0.05.

## 3. Results and Discussion

### 3.1. The Effects of PNO on Anxious–Depressive-Like Behaviors

The management of neuropsychiatric impairments in AD proved to be quite problematic, especially because the evidence base for an appropriate pharmacological approach is limited and ambiguous. Anxious, depressive, and psychotic features, as well as apathy and agitation tend to be unresponsive to acetylcholinesterase inhibitors or memantine in AD cases; hence, antipsychotics, antidepressants, sedatives drugs, or anxiolytics are seen as a rational complementary therapy in AD and frequently prescribed [83]. However, the use of such adjuvant therapy has been associated with a broad spectrum of adverse effects [84,85]. In this study, we assessed the effects of PNO administration on anxious–depressive-like behaviors in the Aβ1-42-induced rat model of AD.

**Figure 2:**
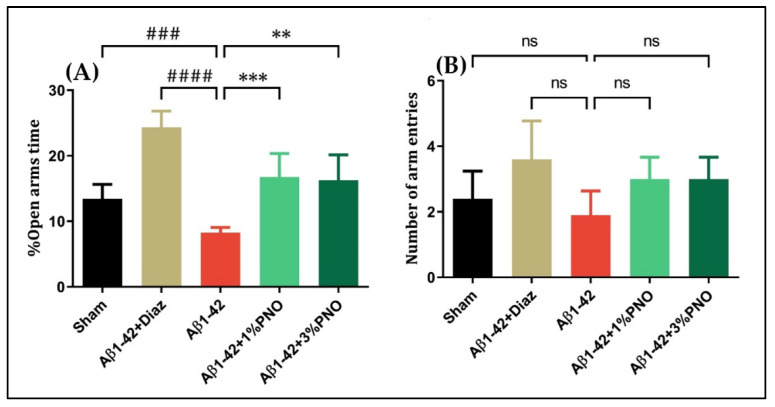
Effects of the inhaled *Pinus halepensis* essential oil (PNO, 1%, and 3%) on the anxious-like behavior in the elevated plus maze test (EPM) in the Aβ1-42-treated rats. Values are means ± S.E.M. (*n* = 10). (**A**) Aβ1-42 vs. sham: ### *p* = 0.0004; Aβ1-42 vs. Aβ1-42 + Diaz: #### *p* < 0.0001; Aβ1-42 vs. Aβ1-42 + 1% PNO: *** *p* = 0.0004 and Aβ1-42 vs. Aβ1-42 + 3% PNO: ** *p* = 0.0012. (**B**) Aβ1-42 vs. sham: ns; Aβ1-42 vs. Aβ1-42 + Diaz: ns; Aβ1-42 vs. Aβ1-42 + 1% PNO: ns; and Aβ1-42 vs. Aβ1-42 + 3% PNO: ns. Diazepam (Diaz, 3 mg/kg) was used as a positive reference drug; ns: non-significant.

The EPM, the gold standard in terms of assessing anxiety-like behaviors, relies on the conflict between a rodent’s preference for protected areas and its innate spontaneous exploratory behavior in novel environments [70], and tracking rodent’s adaptative behavior in the absence of rewards, punishments, or explicit threats [86]. This study showed that single-dose Aβ1-42 administration induced an anxious behavior, reflected by the reduced time spent in the open arms observed in Aβ1-42 pre-treated rats relative to sham-operated rats (*p* = 0.0004) (Figure 2A). However, PNO ameliorated Aβ1-42 anxiogenic effects, significantly increasing the exploratory behavior in the open arms for Aβ1-42 pre-treated rats inhaling 1% PNO (*p* = 0.0004) or 3% PNO, respectively (*p* = 0.0012) (Figure 2A). Regarding locomotor activity within EPM, overall differences between the experimental groups were observed, but the statistical significance was not reached (Figure 2B). Diazepam (Diaz), a potent anxiolytic benzodiazepine [87], was utilized as positive control within the EPM.

**Figure 3:**
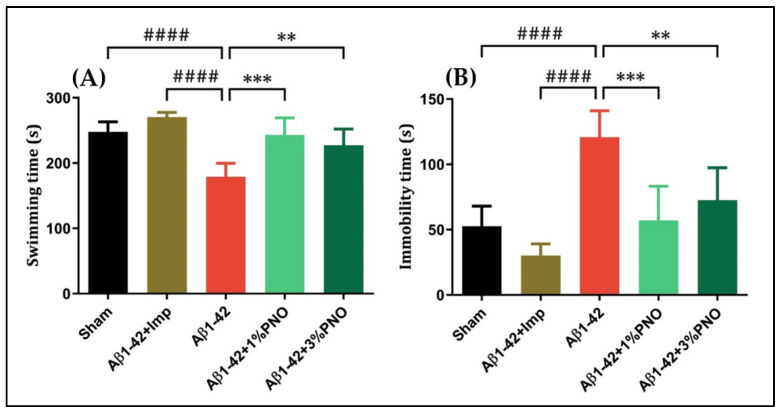
Effects of the inhaled *Pinus halepensis* essential oil (PNO, 1%, and 3%) on the depressive-like behavior in the forced swimming test (FST) in the Aβ1-42-treated rats. Values are means ± S.E.M. (*n* = 10). (**A**) Aβ1-42 vs. sham: #### *p* < 0.0001; Aβ1-42 vs. Aβ1-42 + Imp: #### *p* < 0.0001; Aβ1-42 vs. Aβ1-42 + 1% PNO: *** *p* = 0.0003 and Aβ1-42 vs. Aβ1-42 + 3% PNO: ** *p* = 0.0022. (**B**) Aβ1-42 vs. sham: #### *p* < 0.0001; Aβ1-42 vs. Aβ1-42 + Imp: #### *p* < 0.0001; Aβ1-42 vs. Aβ1-42 + 1% PNO: *** *p* = 0.0001 and Aβ1-42 vs. Aβ1-42 + 3% PNO: ** *p* = 0.0015.

The FST, widely used to assess depressive-like behavior in rodents, is based on the premise that immobility reflects a state of behavioral despair and depression, whereas escape-directed behavior reflects an antidepressant-like state [88]. Herein, it has been observed that single-dose Aβ1-42 administration induced a depressive-like profile, characterized by decreases in swimming time (*p* < 0.0001) (Figure 3A) and increments in immobility time (*p* < 0.0001) (Figure 3B), both changes being statistically significant as compared to sham-operated rats. The inhalation of PNO in both concentrations resulted in significant increases in the swimming time (*p* = 0.0003 for 1% PNO and *p* = 0.0022 for 3% PNO) (Figure 3A) as well as significant reductions of the immobility time (*p* = 0.0001 for 1% PNO and *p* = 0.0015 for 3% PNO) (Figure 3B). Imipramine (Imp), a tricyclic antidepressant, also assessed as a disease-modifying treatment for AD [89], has been used as a positive control within the FST.

The PNO potential to ameliorate anxious–depressive-like behaviors in an Aβ1-42-induced AD model is most likely mediated by the PNO major components, namely *β*-caryophyllene, *α*-pinene, and myrcene [65], especially for all these components anxiolytic and/or antidepressant properties have been already reported. Antidepressant-like effects of *β*-caryophyllene were described in a rat model induced by a chronic restraint stress procedure [90]. *β*-caryophyllene’s potential to ameliorate depressive-like behaviors has been also reported in an experimentally induced diabetes mice model [91]. Moreover, an in vivo study showed that *β*-caryophyllene has anxiolytic effects like those of diazepam [92]. Myrcene has been proven to be another robust anxiolytic agent when acutely administered to zebrafish [93]. Anxiety-relieving properties were demonstrated for *α*-pinene too, in a mice schizophrenia model induced by dizocilpine administration [94]; *α*-pinene is also recommended for management of sleep disorders associated with anxiety [95].

### 3.2. The Effects of PNO on ARC mRNA Level

**Figure 4:**
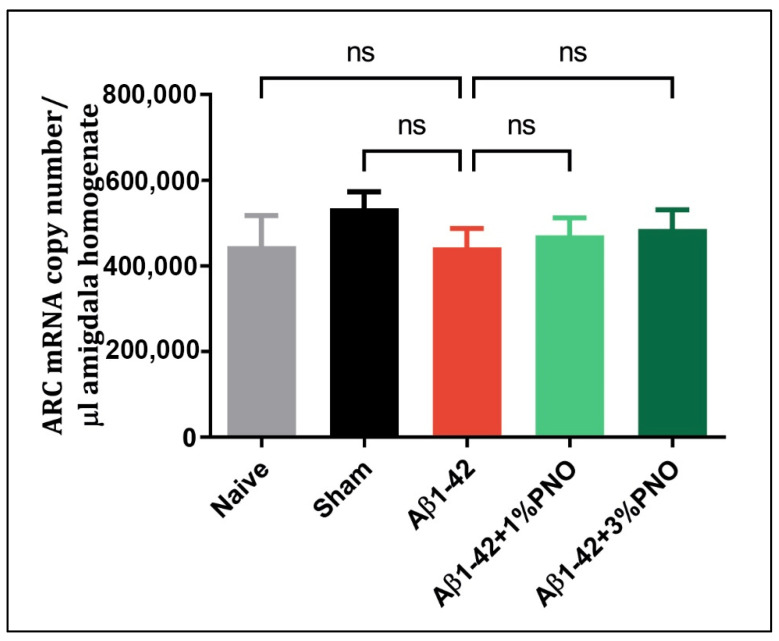
Effects of the inhaled *Pinus halepensis* essential oil (PNO, 1%, and 3%) on the ARC mRNA copy number determined in the rat amygdala homogenates of the Aβ1-42-treated rats. Values are means ± S.E.M. (*n* = 5); ns: non-significant.

*Arc* is a plasticity-related gene, belonging to the immediate–early gene family [96]. Its induction occurs shortly after synaptic activation, being expressed in the excitatory neurons because of behavioral assessments [97]. Although most of the research directions are focused on the link between ARC expression and long-term potentiation (LTP), learning and memory processes [98,99,100], few studies revealed that ARC modulation influences mood-related behaviors, being induced in different brain regions in response to emotionally relevant experiences [101,102,103]. It appears that depression in major depressive disorder [104] and adult anxiety as a consequence of alcoholism during adolescence [105] are characterized by a deficit of ARC expression, yet this seems to not be valid for anxious–depressive behaviors characteristic of AD (Figure 4). Moreover, the anti-anxiety and antidepressive properties of the PNO appear independent of *Arc* gene modulation (Figure 4).

### 3.3. The Effects of PNO on Neuroinflammation—AD-Related

**Figure 5:**
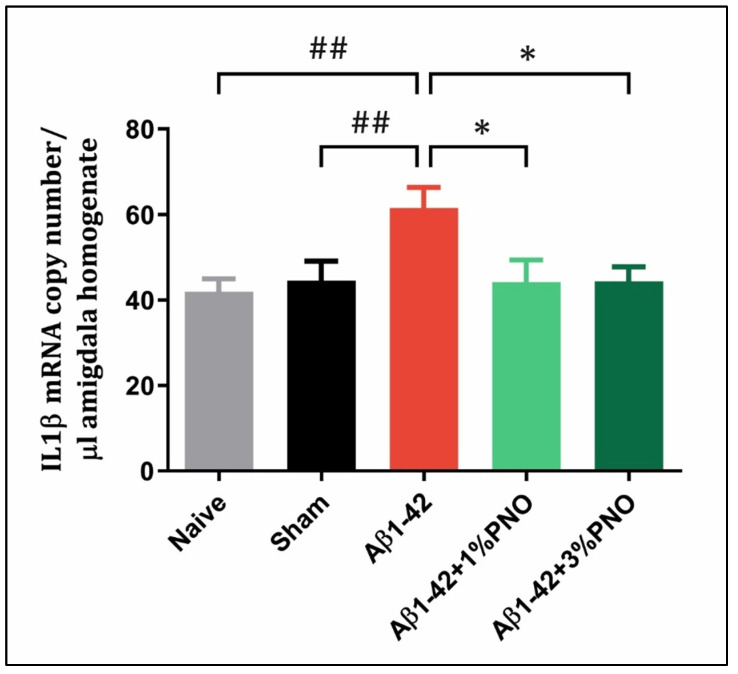
Effects of the inhaled *Pinus halepensis* essential oil (PNO, 1%, and 3%) on the IL-1*β* mRNA copy number determined in the rat amygdala homogenates of the Aβ1-42-treated rats. Values are means ± S.E.M. (*n* = 5). Aβ1-42 vs. Naive: ## *p* = 0.0037; Aβ1-42 vs. sham: ## *p* = 0.0088; Aβ1-42 vs. Aβ1-42 + 1% PNO: * *p* = 0.0109 and Aβ1-42 vs. Aβ1-42 + 3% PNO: * *p* = 0.0204.

Neuroinflammation, as a key contributor to AD development, was reported more than 20 years ago, and different studies reveal that this early disease-aggravating factor starts decades before severe AD-related, cognitive impairments [106,107]. Among different inflammatory pathways, the interleukin-1*β* (IL-1*β*)-signaling pathway has been demonstrated to be directly involved in AD progression, the levels of serum IL-1*β* serving as a stage indicator in the neurodegeneration process [108,109]. The results obtained in the present study are following the existing literature, with Aβ1-42 pre-treated rats being detected as having a significant IL1*β* overexpression as compared to both sham-operated (*p* = 0.0088) and naïve rats (*p* = 0.0037) (Figure 5). However, administration via inhalation of PNO in both concentrations significantly reduced the number of IL1β mRNA copies in the Aβ1-42 pre-treated rats (*p* = 0.0109 for 1% PNO and *p* = 0.0204 for 3% PNO) (Figure 5). It is worth noting that no significant change has been observed between naïve and sham-operated rats regarding the IL1*β* mRNA copy number, which indicates that the neurosurgery did not induce chronic inflammation (*p* = 0.8887) (Figure 5).

The anti-inflammatory properties of PNO may be attributed to its major components, for which neuroprotective properties were revealed in different murine AD-like models. It appears that *β*-caryophyllene reduced neuroinflammation in a transgenic APP/PS1 mice AD model by decreasing the expression of the tumor necrosis factor-α (TNF-*α*) and IL-1*β* proinflammatory cytokines [110]. *α*-pinene countered the inflammation Aβ1-42 associated by modulating TNF-α, IL-1β, and interleukin-6 (IL6) expressions in an AD-like model induced in male Wistar rats [111]. Myrcene’s anti-inflammatory effects, manifested through reduction of TNF-α and IL6, were observed in an AD mouse model induced by aluminum trichloride (AlCl_3_) and D-galactose administration [112].

### 3.4. The Effects of PNO on Oxidative Stress—AD-Related

**Figure 6:**
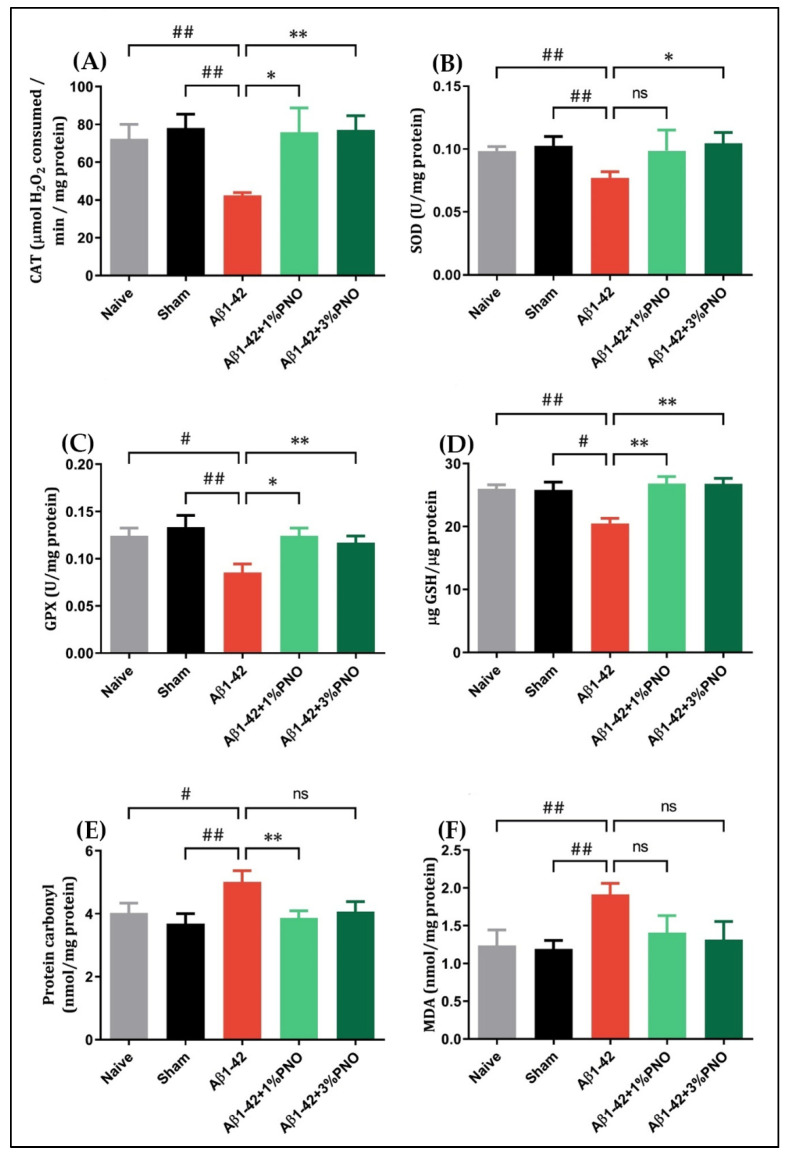
Effects of the inhaled *Pinus halepensis* essential oil (PNO, 1%, and 3%) on (**A**) catalase (CAT), (**B**) superoxide dismutase (SOD), (**C**) glutathione peroxidase (GPX), (**D**) total content of reduced glutathione (GSH), (**E**) protein carbonyl, and (**F**) malondialdehyde (MDA) in the amygdala of Aβ1-42-treated rats. Values are means ± S.E.M. (*n* = 5). (**A**) Aβ1-42 vs. Naive: ## *p* = 0.0025; Aβ1-42 vs. sham: ## *p* = 0.0027; Aβ1-42 vs. Aβ1-42 + 1% PNO: * *p* = 0.0238 and Aβ1-42 vs. Aβ1-42 + 3% PNO: ** *p* = 0.0047. (**B**) Aβ1-42 vs. Naive: ## *p* = 0.0041; Aβ1-42 vs. sham: ## *p* = 0.0066 and Aβ1-42 vs. Aβ1-42 + 3% PNO: * *p* = 0.0364. (**C**) Aβ1-42 vs. Naive: # *p* = 0.0255; Aβ1-42 vs. sham: ## *p* = 0.0027; Aβ1-42 vs. Aβ1-42 + 1% PNO: * *p* = 0.0231 and Aβ1-42 vs. Aβ1-42 + 3% PNO: ** *p* = 0.0034. (**D**) Aβ1-42 vs. Naive: ## *p* = 0.0020; Aβ1-42 vs. sham: # *p* = 0.0103; Aβ1-42 vs. Aβ1-42 + 1% PNO: ** *p* = 0.0053 and Aβ1-42 vs. Aβ1-42 + 3% PNO: ** *p* = 0.0037. (**E**) Aβ1-42 vs. Naive: # *p* = 0.0157; Aβ1-42 vs. sham: ## *p* = 0.0084 and Aβ1-42 vs. Aβ1-42 + 1% PNO: ** *p* = 0.0051. (**F**) Aβ1-42 vs. Naive: # *p* = 0.0357 and Aβ1-42 vs. sham: ## *p* = 0.0084; ns: non-significant.

An ineffective antioxidant defense combined with reactive oxygen species (ROS) overproduction leads to oxidative stress, which is accounted as being responsible for cellular injury in aging and degenerative pathologies [113]. The brain, generally presenting very low antioxidant levels, is particularly susceptible to oxidative damage [114]. In AD, brain regions such as the prefrontal cortex, hippocampus, and amygdala are more vulnerable to oxidative damage, dendritic shrinking, and amygdala hyperactivity specifically triggered by oxidative stress [115]. In the present study, on the amygdala level, it was observed that single-dose administration of Aβ1-42 efficiently induced oxidative state-like changes, such as elevated protein oxidation (*p* = 0.0084) (Figure 6E) and lipid peroxidation (*p* = 0.0084) (Figure 6F) rates, as well as a reduced level of GSH (*p* = 0.0103) (Figure 6D) and decreased activity of antioxidant enzymes CAT (*p* = 0.0027) (Figure 6A), SOD (*p* = 0.0066) (Figure 6B), and GPX (*p* = 0.0027) (Figure 6C). The inhaled PNO in both concentrations, however, restored antioxidant defense, significantly elevating CAT (*p* = 0.0238 for 1% PNO and *p* = 0.0047 for 3%PNO) (Figure 6A) and GPX (*p* = 0.0231 for 1% PNO and *p* = 0.0034 for 3% PNO) (Figure 6C) activity as well as the level of GSH (*p* = 0.0053 for 1% PNO and *p* = 0.0037 for 3% PNO) (Figure 6D) in Aβ1-42 pre-treated rats. Regarding SOD, its activity has been significantly increased only in Aβ1-42 pre-treated rats inhaling 3% PNO (*p* = 0.0364) (Figure 6B). PNO inhalation also reduced aberrant protein oxidation in Aβ1-42 pre-treated rats, but only when administered at a 1% concentration were the induced changes statistically significant (*p* = 0.0051) (Figure 6E). When referring to lipid peroxidation, although PNO reduced MDA levels in Aβ1-42 pre-treated rats, the induced modifications did not reach statistical significance (Figure 6F).

Targeting neuronal oxidative stress via therapeutics capable of not only passively scavenging radicals but also interfering with signal transduction pathways arose as a satisfying pharmacological approach in neurodegenerative disorders [116,117]. *α*-pinene demonstrated properties to combat AD-related oxidative stress through modulation of NF-E2-related factor 2/Kelch-like ECH-associated protein 1/antioxidant response element (Nrf2/Keap1/ARE) and interconnected pathways [118,119]. Myrcene appears to manifest its antioxidant properties by upregulating Nrf2 transcription factor gene expression, as well as the expression of its target genes CAT, SOD1, GPX1, GSTP1, NQO1, GSR, and HMOX1 [120]. Antioxidant and neuroprotective effects of *β*-caryophyllene seem to be linked to G-protein-coupled type 2 cannabinoid receptor (CB2R)-dependent Nrf2/heme oxygenase-1 (HO-1) antioxidant axis activation and inhibition of the HMG-CoA reductase activity [121,122,123].

### 3.5. The Effects of PNO on Cell Death—AD-Related

**Figure 7:**
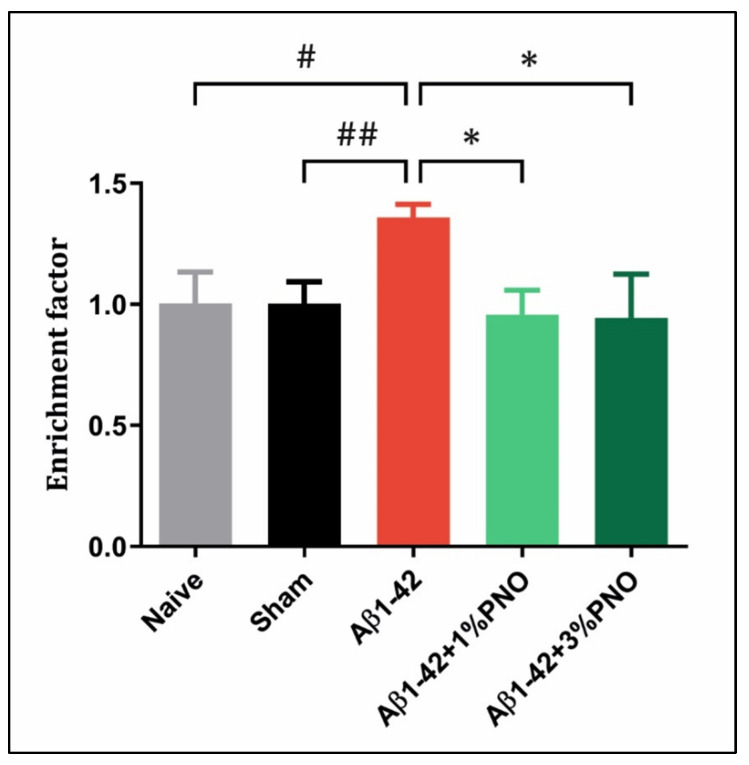
Effects of the inhaled *Pinus halepensis* essential oil (PNO, 1%, and 3%) on apoptotic state determined in the rat amygdala homogenates of the Aβ1-42-treated rats. Values are means ± S.E.M. (*n* = 5). Aβ1-42 vs. Naive: # *p* = 0.0229; Aβ1-42 vs. sham: ## *p* = 0.0026; Aβ1-42 vs. Aβ1-42 + 1% PNO: * *p* = 0.0129 and Aβ1-42 vs. Aβ1-42 + 3% PNO: * *p* = 0.0265.

The gradual cell death detected in AD has been historically attributed to tau hyperphosphorylation and Aβ-aberrant aggregation, although the exact cell death pathway responsible for neuronal loss in AD has not been yet clearly established [124,125]. Apoptosis, one of the best mechanistically described programmed cell death pathways, is characterized by chromatin condensation, nuclear fragmentation, caspase activation, and the formation of apoptotic bodies [125]. Histone release has been highly associated with DNA fragmentation during the apoptotic process, and mounting evidence suggests that core histones, such as H2A, H2B, H3, and H4, as well as link histone (H1), detach from genomic DNA, translocate into the cytoplasm, and subsequently release into the extracellular space [126]. In the present study, significantly increased DNA fragmentation has been detected in the Aβ1-42 pretreated rats as compared to both sham-operated (*p* = 0.0026) and naïve rats (*p* = 0.0229) (Figure 7). However, the administration of PNO in both concentrations significantly antagonized Aβ1-42 effects, reducing DNA fragmentation in the amygdala of Aβ1-42 pretreated rats (*p* = 0.0129 for 1% PNO and *p* = 0.0265 for 3% PNO) (Figure 7).

The knowledge involving PNO’s main component effects regarding DNA fragmentation and cell death in AD is scarce. However, it appears that both *α*-pinene and myrcene presented neuroprotective properties against apoptosis in different animal models of cerebral ischemia [127,128]. On the other hand, *β*-caryophyllene showed anti-apoptotic properties in an in vitro model of Parkinson’s disease [129].

## 4. Conclusions

**Figure 8:**
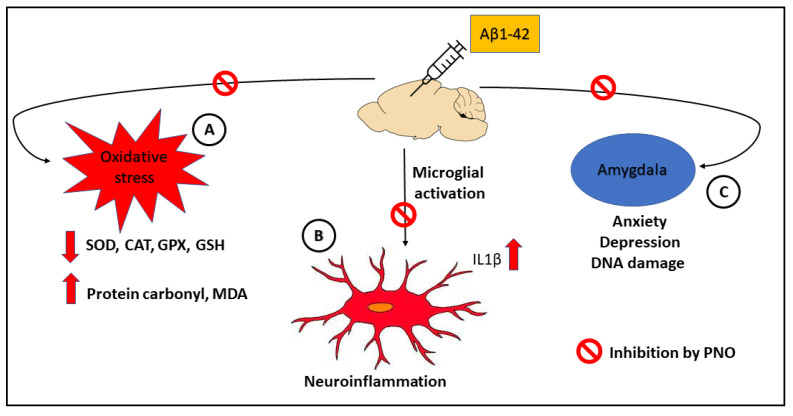
The neuroprotective effects of *Pinus halepensis* essential oil (PNO) against Aβ1-42-induced neuronal dysfunction in the rat amygdala. (**A**) PNO may be able to decrease the oxidative stress level in the rat amygdala by increasing the antioxidant enzymes activity and reducing the protein oxidation (protein carbonyl) and lipid peroxidation (MDA) levels. (**B**) PNO can reduce the level of gliosis (neuroinflammation) in the rat amygdala. (**C**) PNO decrease anxiety–depresive-like behaviors and DNA damage in the rat amygladala.

The present experimental study has been designed to assess the anxiolytic and antidepressive properties of PNO in a rodent Aβ-induced AD model. Altogether, the obtained results provide evidence that PNO attenuates Aβ-related neuropsychiatric impairments, such as anxiety and depression, although these improvements appear to not be mediated via ARC modulation. Moreover, PNO restored the antioxidant defense system, upregulating antioxidant enzyme activity and reducing protein carbonylation at the amygdala level. In addition, PNO ameliorated both neuroinflammation and neuroapoptosis by downregulating IL1*β* gene expression and decreasing DNA fragmentation in the amygdalas of rats bearing AD-like neuropsychiatric deficits. Overall, based on the present findings, it can be concluded that PNO acts as potent multi-functional anti-AD agent. Finally, the biological mechanism by how PNO alleviate Aβ-induced neuronal dysfuctions can be summarized in Figure 8.

## Data Availability

The data presented in this study are available on request from the corresponding author.
